# Comprehensive Assessment of Initial Adaptation of Extended-Spectrum β-Lactamase–Positive ST131 *Escherichia coli* to Carbapenem Exposure

**DOI:** 10.1093/infdis/jiae587

**Published:** 2024-11-27

**Authors:** William C Shropshire, Xinhao Song, Jordan Bremer, Seokju Seo, Susana Rodriguez, Selvalakshmi Selvaraj Anand, An Q Dinh, Micah M Bhatti, Anna Konovalova, Cesar A Arias, Awdhesh Kalia, Yousif Shamoo, Samuel A Shelburne

**Affiliations:** Department of Infectious Diseases and Infection Control, The University of Texas MD Anderson Cancer Center; Department of Biosciences, Rice University; Department of Infectious Diseases and Infection Control, The University of Texas MD Anderson Cancer Center; Department of Biosciences, Rice University; Department of Microbiology and Molecular Genetics, McGovern Medical School, The University of Texas Health Science Center at Houston; Program in Diagnostic Genetics and Genomics, The University of Texas MD Anderson Cancer Center School of Health Professions; Division of Infectious Diseases and Center for Infectious Diseases Research, Houston Methodist Hospital and Houston Methodist Research Institute; Department of Laboratory Medicine, The University of Texas MD Anderson Cancer Center, Houston; Department of Microbiology and Molecular Genetics, McGovern Medical School, The University of Texas Health Science Center at Houston; Division of Infectious Diseases and Center for Infectious Diseases Research, Houston Methodist Hospital and Houston Methodist Research Institute; Department of Medicine, Weill Cornell Medical College, New York, New York; Program in Diagnostic Genetics and Genomics, The University of Texas MD Anderson Cancer Center School of Health Professions; Department of Biosciences, Rice University; Department of Infectious Diseases and Infection Control, The University of Texas MD Anderson Cancer Center; Program in Diagnostic Genetics and Genomics, The University of Texas MD Anderson Cancer Center School of Health Professions; Department of Genomic Medicine, The University of Texas MD Anderson Cancer Center, Houston

**Keywords:** non-carbapenemase carbapenem resistance, ESBL gene amplification, experimental evolution, pseudo compound transposon, sequence type 131

## Abstract

**Background:**

It remains unclear how high-risk *Escherichia coli* lineages, like sequence type (ST) 131, initially adapt to carbapenem exposure in their progression to carbapenem resistance.

**Methods:**

Carbapenem mutation frequency was measured in multiple subclades of extended-spectrum β-lactamase (ESBL)–positive ST131 clinical isolates using a fluctuation assay followed by whole genome sequencing (WGS) characterization. Genomic, transcriptomic, and porin analyses of the ST131 C2/*H*30Rx isolate MB1860, under prolonged, increasing carbapenem exposure was performed using 2 experimental evolutionary platforms to measure fast versus slow adaptation.

**Results:**

All 13 ESBL-positive ST131 strains selected from a diverse (n = 184) ST131 bacteremia cohort had detectable ertapenem (ETP) mutational frequencies, with a positive correlation between initial ESBL gene copy number and mutation frequency (*r* = 0.87, *P* < 1e-5). WGS analysis of mutants showed that initial response to ETP exposure resulted in significant increases in ESBL gene copy numbers or mutations in Omp genes in the absence of ESBL gene amplification with subclade-specific associations. In both experimental evolutionary platforms, MB1860 responded to initial ETP exposure by increasing *bla*_CTX-M-15_ copy numbers via modular, IS*26*-mediated pseudocompound transposons (PCTns). Increased transcript level of genes present within the PCTn was a conserved expression signal in both experimental evolutionary platforms. Stable mutations in Omp encoding genes were detected only after prolonged increasing carbapenem exposure, consistent with clinical observations.

**Conclusions:**

ESBL gene amplification is a conserved response to initial carbapenem exposure, especially within the high-risk ST131 C2/*H*30Rx subclade. Targeting such amplification could assist with mitigating carbapenem resistance development.

## Teaser

High-risk ST131 *Escherichia coli* strains, particularly C2/*H*30Rx subclades, adapt to initial carbapenem exposure by insertion sequence–mediated extended-spectrum β-lactamase gene amplification. This gene amplification may precede stable mutations in porin genes, potentially leading to resistance with manageable fitness costs.

The increased worldwide prevalence of extended-spectrum β-lactamase (ESBL)–producing *Escherichia coli* (ESBL-*Ec*) represents a serious public health challenge, significantly contributing to the global burden of antimicrobial resistance (AMR)–related mortality [1]. A recent population-based United States (US) study indicated that from 2012 to 2017 there was a 53.3% increase in ESBL-producing Enterobacterales infections, with 87% of that increase due to ESBL-*Ec* infections [2]. Carbapenems are recommended for treating complicated ESBL-*Ec* infections [3], yet carbapenem resistance can still develop within these pathogens that lack carbapenem-degrading carbapenemases, driven by increased ESBL enzyme activity and altered outer membrane permeability that reduces intracellular carbapenem levels [4–6]. While these non-carbapenemase-producing, carbapenem-resistant *E coli* (non-CP-CR*Ec*) mechanisms are becoming more well understood, there remains limited knowledge of how ESBL-*Ec* strains initially adapt to carbapenem exposures prior to acquiring clinically defined carbapenem resistance.

Perhaps the most successful multidrug-resistant *E coli* lineage of the 21st century is the extraintestinal pathogenic sequence type (ST) known as ST131 [7–9]. Given this lineage's global dissemination and wide range of observed AMR phenotypes, ST131 has been considered an excellent model for examining AMR evolutionary trajectories [10–12]. Although there is significant spatiotemporal heterogeneity in population structure of carbapenem-resistant *E coli* [13, 14], a recent population-based US surveillance study of carbapenem-resistant Enterobacterales found that ST131 was the leading cause of carbapenem-resistant *E coli* infections with the vast majority having non-CP-CR*Ec* mechanisms [15].

Given the substantial prevalence of ST131 causing non-CP-CR*Ec* infections, we sought to perform a comprehensive assessment of ESBL-positive ST131 isolates’ initial adaptation to carbapenems using a multidisciplinary approach. Our study on ST131 bacteremia strains, particularly C2/*H*30Rx subclades, reveals that insertion sequence (IS)–mediated ESBL gene amplification is a key driver of initial carbapenem resistance. This response across multiple evolutionary models highlights a potential target for reducing resistance progression.

## MATERIALS AND METHODS

### Data Collection

ST131 *E coli* bacteremia events were abstracted from our REDCap database and cross-referenced with MicroBank isolates saved at −80°C in 20% glycerol stocks. Whole genome sequencing (WGS) results of *E coli* bacteremia isolates identified as ST131 from previous studies were included in the study [4, 5, 16–18].

### Antimicrobial Susceptibility Testing

Clinical isolates were tested for their minimum inhibitory concentrations (MICs) to ertapenem (ETP) and meropenem (MEM) using both gradient strip Etest as well as broth microdilution following Clinical and Laboratory Standards Institute M100 (2022) guidelines [19].

### Modified Luria Delbruck Assay

A modified fluctuation assay was performed for the purposes of measuring mutation frequency rate in our samples as previously described [20, 21]. Detail on assay and mutation frequency calculation is included in the Supplementary Material. For samples that had detectable positive growth, up to 4 mutants were randomly selected for ETP MIC determination using broth microdilution as previously described above and stored at −80°C for future genomic characterization as described below.

### Microfluidic System and Flask Transfer Experimental Evolutionary Platform Protocols

The C2*H*30Rx strain MB1860 [4] was passaged using 2 distinct experimental evolutionary platforms. The novel microfluidic system (MFS) methodology has been published previously [22]. Further detail on the novel MFS system and the flask transfer protocol (FTP) methodology are included in the Supplementary Material. Daily populations as well as end point isolates (EPIs) collected from colonies streaked out from populations with last ETP exposure were collected for further characterization, described below.

### DNA Sequencing

Mutants collected from our modified fluctuation assay of ST131 subclades (n = 52) and daily MB1860 population isolates collected from experimental evolution platforms (n = 64), respectively, were collected for Illumina short-read sequencing. Samples underwent genomic DNA extraction and Illumina short-read, paired-end sequencing DNA library prep as previously described [5]. A total of 4 samples (MB6054 [n = 4]; MB10029 [n = 1]) from the modified fluctuation assay were censored due to paired-end read contamination, leaving a total of 48 samples available for mutant analysis.

### RNA Sequencing

Daily MB1860 population isolates from both experimental evolution platforms (ie, FTP and MFS) were selected for RNA sequencing (RNA-Seq). In particular, we selected daily populations that were exposed to approximately 1 × ETP MIC = 0.0625 μg/mL (ie, FTP population 1, day 2 isolates [FTP-P1-D2] and MFS population 1, day 26 [MFS-P1-D26] isolates; we collectively refer to these isolates as “experiment”), as well as passaged isolates collected from the same founder population and same day with no ETP exposure from each respective population (ie, FTP-P1-D2 and MFS-P1-D26 without ETP exposure). Three technical replicates from each group were grown to mid-log phase (optical density at 600 nm [OD_600_] = 0.5 ± 0.05) with the experimental isolates grown in Luria-Bertani broth supplemented with 0.03 μg/mL ETP whereas control isolates were grown without ETP exposure. Further detail on RNA-Seq methodology is included in the Supplementary Material.

### Western Blot Analysis

Immunoblot analysis of outer membrane porins has been described previously [23]. In brief, samples are grown to mid-log phase and then standardized by OD_600_. Samples are then boiled and subjected to electrophoresis on sodium dodecyl sulfate polyacrylamide gel supplemented with 4M urea. Anti-rabbit antibodies targeting OmpC/OmpF/OmpA have been previously validated and reported [24, 25].

### Comparative Genomics

A total of 183 ST131 *E coli* paired-end short-reads that were sequenced from previous molecular epidemiology projects [4, 5, 16–18] were aligned to MB1860 (RefSeq number NZ_CP049085.2) using the Snippy-v4.6.0 variant call pipeline (T. Seemann, https://github.com/tseemann/snippy). Core genome alignment file was used to infer a recombination-free maximum likelihood phylogenetic tree using UFBoot2 (1000X) and ModelFinder with IQTree-2-v2.0.3 [26] and Gubbins-v3.2.1 [27]. Further comparative genomics analysis detail can be found in the Supplementary Material.

### Differential Expression Analysis

MB1860-passaged isolates were selected in the MFS and FTP system with and without ETP selective pressure (passage control, 0 µg/mL ETP; experiment, 0.0625 µg/mL ETP). A full description of differential expression and downstream RNA-seq analysis can be found in the Supplementary Material.

### Statistical Analysis

All statistical analysis was performed in R version 4.3.2 software as previously described [5, 17, 18].

## Supplementary Material

jiae587_Supplementary_Data

## Figures and Tables

**Figure 1.**
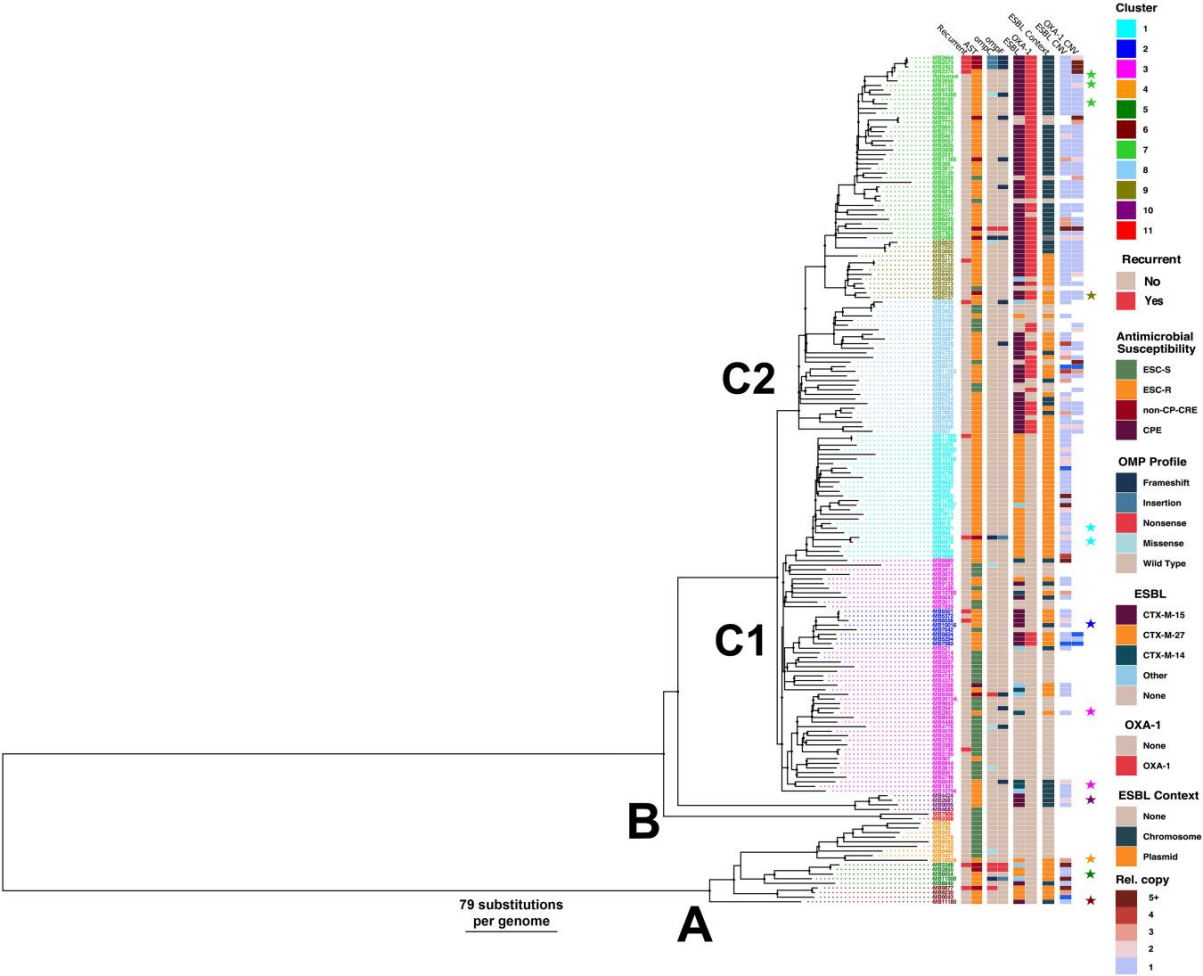
Population structure of sequence type (ST) 131 *Escherichia coli* bacteremia isolates. Midpoint rooted, recombination-masked, core genome–inferred maximum-likelihood phylogeny of ST131 *E coli* (n = 184) collected from 2013 to 2020 at our institution. The 3 major subclones with clade C split into C1 and C2 are labeled on the dendrogram. Terminal branch tips are colored by subcladal structure (n = 11) as identified using a Bayesian analysis of population structure. Columnar metadata are labeled with corresponding categories presented in the figure key at right. Thirteen ST131 isolates from 9 subclades that were selected for carbapenem mutation frequency analysis are labeled by stars that appear to the right of columnar metadata. Abbreviations: AST, antimicrobial susceptibility testing; CNV, copy number variant; CPE, carbapenemase-producing Enterobacterales; ESBL, extended-spectrum β-lactamase; ESC-R, extended-spectrum cephalosporin resistant; ESC-S, extended-spectrum cephalosporin susceptible; non-CP-CRE, non-carbapanemase-producing carbapenem resistant Enterobacterales.

**Table 1.** Carbapenem Fluctuation Assay Results for Select Sequence Type 131 Extended-Spectrum β-Lactamase–Positive Strains

Sample	Clade (Sublevel)	ESBL	ESBL Context	P_0_ ESBL CNV	ETP Fluctuation Assay Results			
					P_0_ ETP MIC (μg/mL)^a^	P_0_ Mutation Frequency^b^	P_1_ ETP MIC (μg/mL)^a^	P_1_ ETP MIC Fold Change (×)
MB10029	A (4)	CTX-M-27	Plasmid	2.97	0.094	12 854	0.25	3
MB6054	A (5)	CTX-M-27	Plasmid	1.42	0.063	564	0.25	4
MB11180	A (6)	CTX-M-15	Chromosome	0.93	0.5	967	1.5	3
MB2681	B0 (10)	CTX-M-15	Chromosome	1.69	0.25	2879	0.5	2
MB10016	C1 (2)	CTX-M-15	Chromosome	1.01	0.094	106	0.5	5
MB1341	C1 (3)	CTX-M-14	Chromosome	1.13	0.25	998	0.5	2
MB2007	C1 (3)	CTX-M-14	Plasmid	1.23	0.063	117	0.5	8
MB2951	C1-M27 (1)	CTX-M-27	Plasmid	1.67	0.094	6874	1.0	11
MB6910	C1-M27 (1)	CTX-M-27	Plasmid	1.47	0.063	749	0.25	4
MB1159	C2 (7)	CTX-M-15	Chromosome	1.06	0.13	1368	1	8
MB1860	C2 (7)	CTX-M-15	Chromosome	0.93	0.25	2242	1	4
MB8420	C2 (7)	CTX-M-15	Chromosome	0.85	0.25	2031	1	4
MB5127	C2 (9)	CTX-M-15	Plasmid	1.37	0.5	1149	1	2

Abbreviations: CNV, copy number variant; ESBL, extended-spectrum β-lactamase; ETP, ertapenem; MIC, minimum inhibitory concentration; P_0_, ancestral clinical strain; P_1_, mutant progeny strain.

^a^MICs are reported as the median of 2 or more repeat experiments performed in duplicate.

^b^Mutation frequencies are reported as the mean number of mutants per 10^8^ cells (n = 3 replicates).

**Figure 2.**
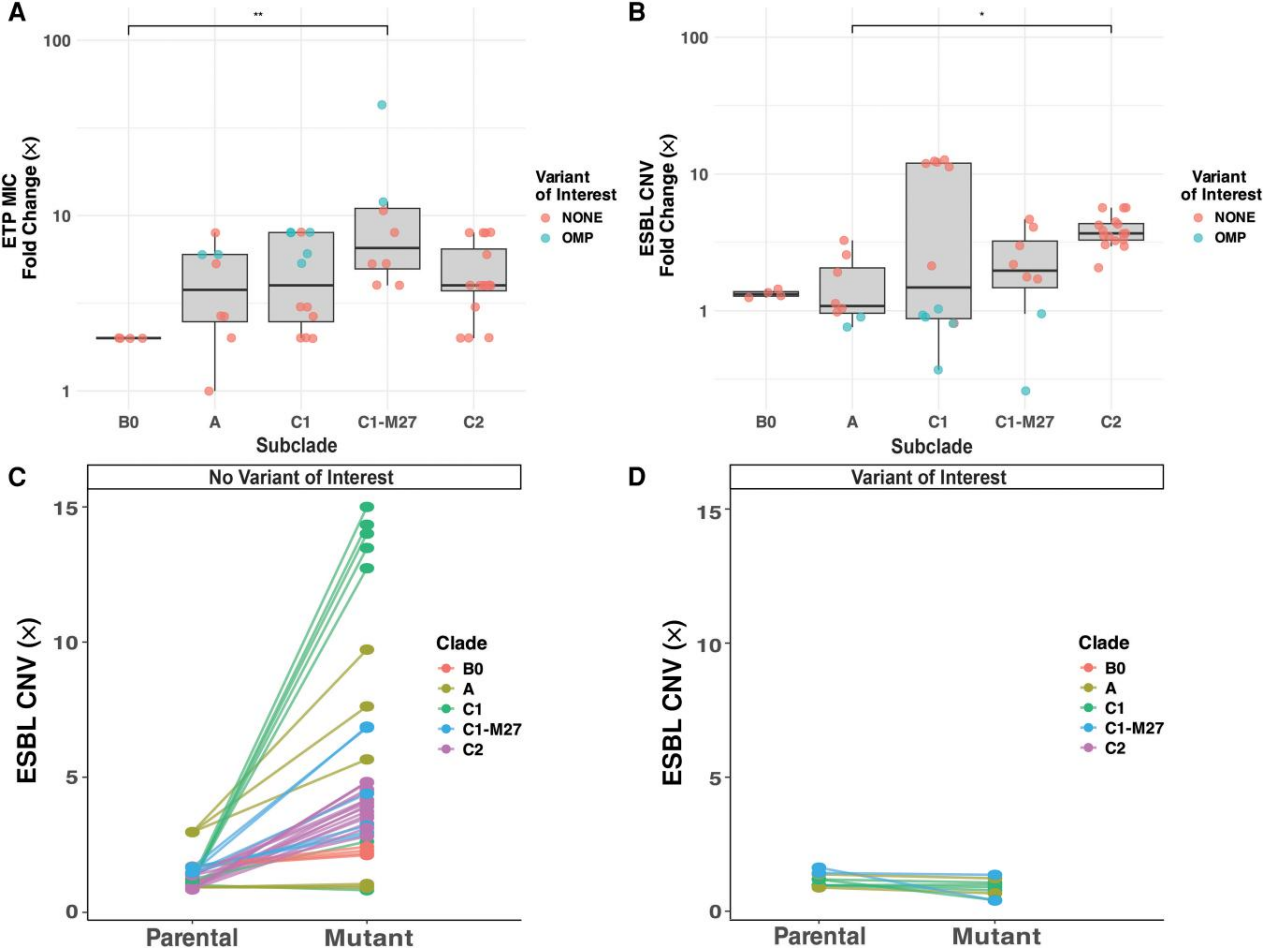
Sequence type (ST) 131 fluctuation assay mutant genomic characterization. Ertapenem (ETP) minimum inhibitory concentration (MIC) fold change (×) (*A*) and extended-spectrum β-lactamase (ESBL) gene fold change (×) (*B*) across 5 predominant subclades of ST131. The y-axis in each respective subpanel is presented in log_10_ scale without data transformation. Variant status is labeled by color as presented in the figure key. ESBL copy number variant (CNV) between parental (ie, carbapenem naive) and progeny mutant (ie, positive growth following ETP exposure) strain stratified by isolates with no variant of interest (*C*) versus strains with a variant of interest detected (*D*), with clade indicated by color in the key. Dunn multiple-comparisons test: **P < .01; *P < .05.

**Figure 3.**
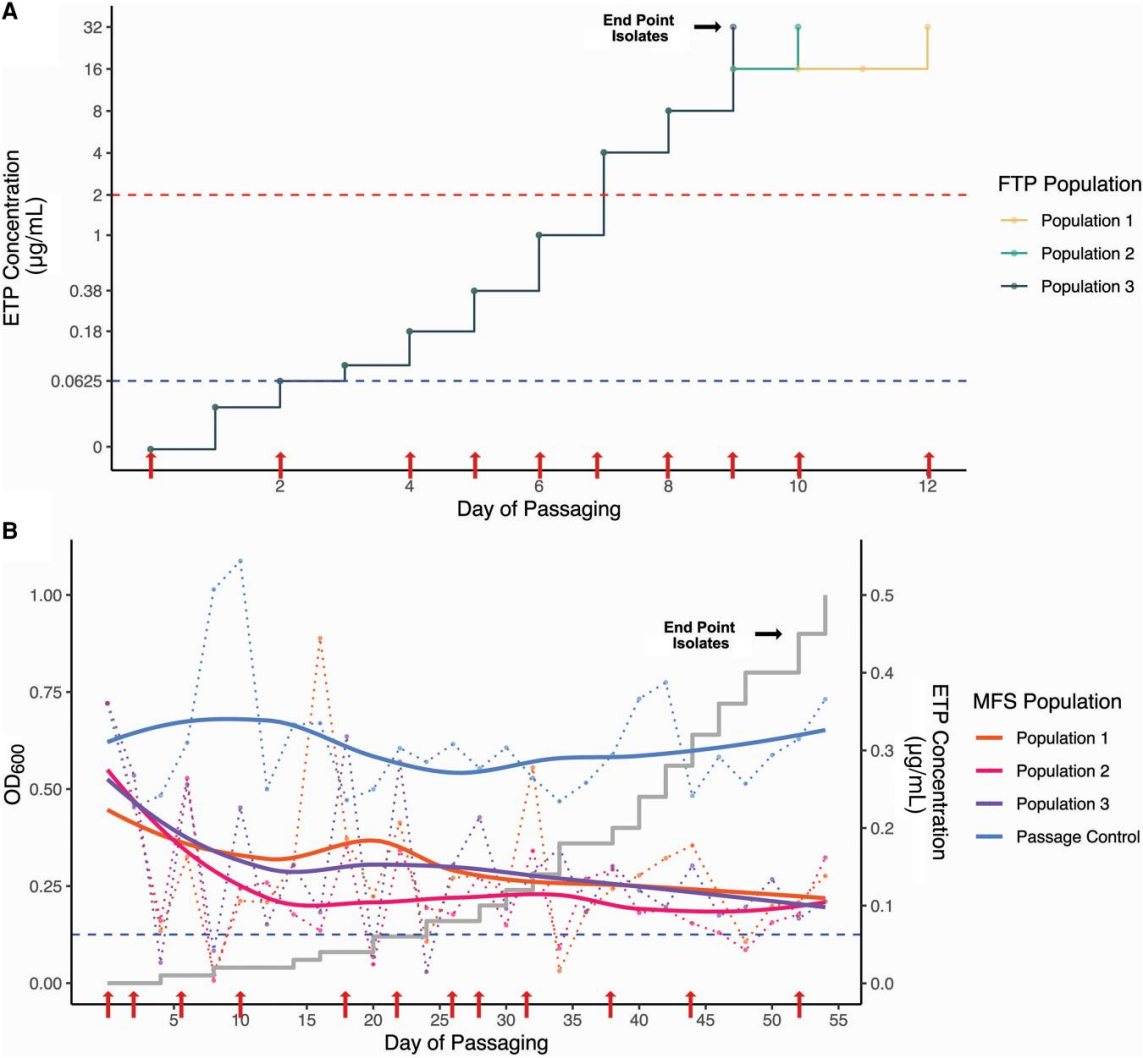
Growth of MB1860 with increasing ertapenem (ETP) concentration gradients. *A*, Flask transfer protocol (FTP) populations (n = 3) exposed to stepwise gradient increase in ETP concentrations with indication of positive growth (optical density at 600 nm [OD_600_] >0.5) at each respective day. *B*, Note that population 1–3 stepwise growth followed comparable trajectories up to day 9 of passaging microfluidic system (MFS) populations (n = 3) with OD_600_ measurements indicated on the left y-axis and ETP concentration stepwise increments (gray solid line) indicated on the right y-axis. Dotted lines indicate variance in OD_600_ measurements over time, with each population color-coded in the key. A fitted loess curve (solid color-coded lines) for OD_600_ measurements of MFS populations is juxtaposed across the line graph. Blue horizontal dotted line indicates ancestral MB1860 ETP minimum inhibitory concentration (0.0625 μg/mL). Red horizontal dotted line (*A*) indicates Clinical and Laboratory Standards Institute carbapenem resistance breakpoint [19]. Red arrows indicate whole genome sequencing sampling timepoints.

**Figure 4.**
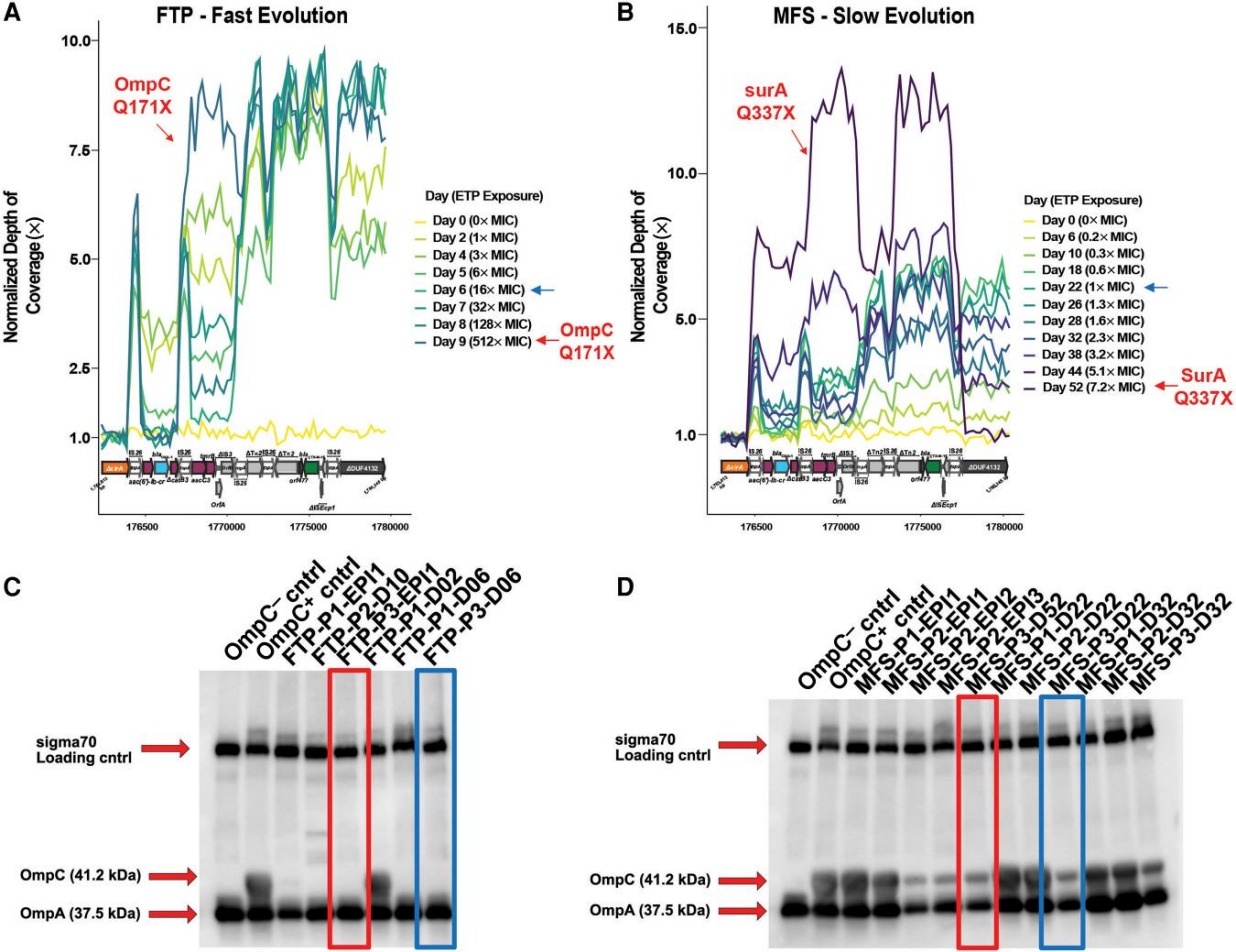
Amplification of the MB1860 pseudocompound transposon (PCTn) and Western blot analysis of outer membrane porins (OMPs) in flask transfer protocol (FTP) and microfluidic system (MFS) experiments. FTP (*A*) and MFS (*B*) normalized coverage depth of MB1860 PCTns indicating amplification of β-lactamases *bla*_OXA-1_ (blue open reading frame) and *bla*_CTX-M-15_ (green open reading frame) as presented on the x-axis. Red arrows indicate where nonsense mutations were detected corresponding with each particular day. FTP and MFS population 3 (P3) isolates are illustrated here as representatives of their respective evolutionary trajectories. A large-scale figure of the MB1860 PCTn shown in relation to the *ompC* gene is presented in Supplementary Figure 2. FTP (*C*) and MFS (*D*) immunoblot analysis of OmpC across select daily population and end point isolates (EPIs). “OmpC^–^ control” is the previously characterized OmpC-negative strain MB9877 [5] and “OmpC^+^ control” is the positive control, parental MB1860 strain. Red and blue boxes correspond to red and blue arrows in (*A*) and (*B*), respectively, highlighting strains of interest.

**Figure 5.**
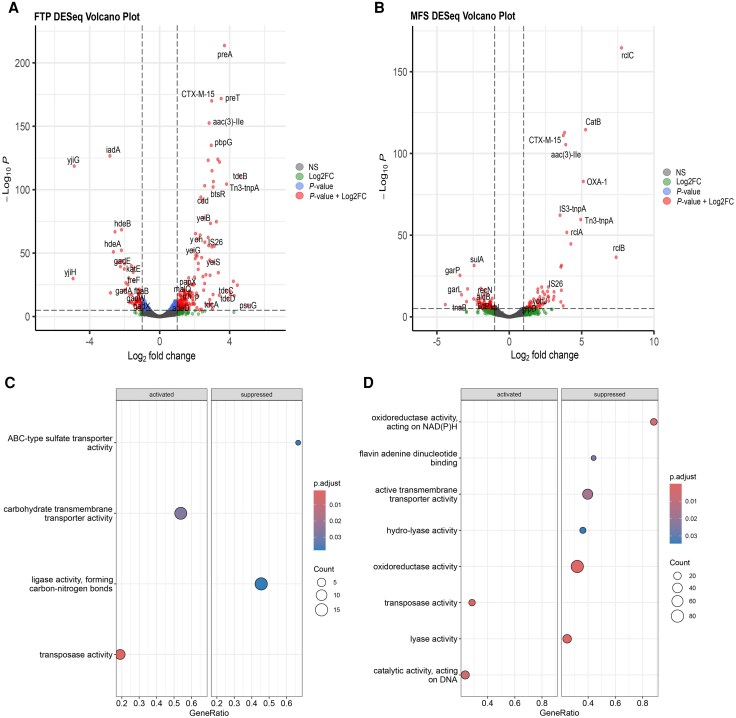
Transcriptome analysis of MB1860 daily population isolates in flask transfer protocol (FTP) and microfluidic system (MFS) platforms. Differential expression analysis was conducted on daily population isolates (FTP population 1, day 2 isolates and MFS population 1, day 26 isolates) and exposed to 0.5× ertapenem (ETP) minimum inhibitory concentration (ie, 0.03 µg/mL ETP) compared to passage controls without ETP exposure. Volcano plots of differentially expressed genes in the FTP (*A*) and MFS (*B*) platforms with genes of interest identified. Dotted lines demarcate genes that have an adjusted *P* value cutoff of 1e-6 and log_2_ fold change = 2, with red dots indicating statistically significant results with absolute log_2_ fold change (FC) values >2. Gene set enrichment analysis of FTP (*C*) and MFS (*D*) platforms with activated vs suppressed gene sets. Gene ratio indicates total fraction of genes belonging to that particular gene set with statistically upregulated or downregulated expression and count labeled in the key, respectively.

## Data Availability

All R scripts used for this analysis can be found in the Figshare published repository (doi: 10.6084/m9.figshare.26400901). WGS data have been made publicly available through the National Institutes of Health BioProject repository PRJNA1141466 and GEO Series accession number GSE273456.

## RESULTS

### The Majority of ST131Non-CP-CR*Ec* Isolates Are Detected Within the C2/*H*30Rx Subclade

There was a total of 184 ST131 bacteremia isolates from 171 unique patients (ie, 13 cases of recurrence; Supplementary Table 1). Figure 1 illustrates the core ST131 genome population structure with the previously designated 3 major subclones labeled. The vast majority of isolates (160/184 [87%]) were from the worldwide disseminated “C clade,” often designated as *H*30R [28] due to its strong association with the *fimH30* allele and fluoroquinolone resistance [29]. There were 11 clusters of ST131 clades identified using a hierarchical Bayesian analysis of the core genome as represented by branch tip color (Figure 1). Carbapenem resistance was detected in at least 1 ST131 bacteremia episode in 11 of 171 patients (6%), with 3 patients infected by strains that converted from being cephalosporin resistant, carbapenem susceptible to a non-CP-CR*Ec* phenotype (Supplementary Table 1). Most carbapenem-resistant isolates (10/11 [91%]) were non-CP-CR*Ec*. Half of all non-CP-CR*Ec* isolates were detected within the cluster 7 C2/*H*30Rx subclade (n = 5) with 80% co-harboring *bla*_CTX-M-15_ and *bla*_OXA-1_ as detailed in Figure 1. The second most commonly detected ESBL-encoding gene detected within non-CP-CR*Ec* isolates was *bla*_CTX-M-27_ (n = 3). When characterizing variants across the 10 non-CP-CR*Ec* isolates (Supplementary Table 2), the only genes with disruptive mutations detected across 2 or more isolates were the outer membrane porin encoding genes *ompC* and *ompF* as well as the transcriptional regulator encoding gene *soxR*, which can negatively regulate efflux activity [30].

### ESBL-Positive ST131Bacteremia Isolates Increase ESBL Gene Copy Number in Response to Initial Ertapenem Exposure Across Multiple ST131 Subclade Backgrounds

To gain insight into the initial ESBL-*Ec* adaptation to carbapenems, we selected 13 carbapenem-susceptible ESBL-positive ST131 isolates from differing subclade backgrounds and measured mutation frequency following exposure to ETP using a fluctuation assay. We observed no mutants above the limit of detection for 3 ESBL-negative ST131 isolates (Supplementary Table 3). All 13 ESBL-positive isolates had detectable mutation frequencies ranging from 106 to 12 854 mutants per 10^8^ cells (Table 1). A strong positive linear relationship was detected between initial ESBL gene copy number in the parental isolate and subsequent mutation frequency calculated (Pearson correlation coefficient: *r* = 0.88; *P* < .001). We selected a subset of ST131 isolates (n = 6) to measure their MEM mutation frequency and detected a significantly narrower range from 69 to 381 mutants per 10^8^ cells, suggesting a smaller adaptive mutation supply for MEM in contrast to ETP (Supplementary Table 4).

We characterized 3 to 4 mutants per parental strain and found that the median ETP MIC fold change was elevated and generally similar across subclades with the exception of a statistically significant difference between the B0 and C1-M27 subclades (Figure 2*A*). When focusing on mutant ESBL gene fold changes relevant to parental strain, there was a range across ST131 subclades, with the C2/*H*30Rx mutants having the greatest increase (median, 3.7× [interquartile range {IQR}, 1.1×]) and A subclade mutants having only a minimal increase (median, 1.1× [IQR, 1.1×]) (Figure 2*B*). Variant calling analysis of 48 mutant strains indicated that the majority did not have single-nucleotide polymorphisms detected relative to parental isolates (median, 0 [IQR, 1]) (Supplementary Figure 1). Nineteen percent (9/48) had a variant in genes known to affect outer membrane permeability (Supplementary Table 5) including *ompC*, *ompF*, *rseA* (ie, gene that encodes anti-sigma-E factor known to regulate *ompF* expression [31]), and *surA*, which encodes a chaperone protein that is responsible for proper folding of outer membrane proteins [32, 33]. When comparing ESBL gene amplification stratified by variant status, mutants that had a variant of interest (ie, mutation in gene that involves outer membrane permeability) had statistically lower ESBL gene fold changes (median, 0.9× [IQR, 0.2×]) compared to isolates without a variant detected (median, 3.3× [IQR, 2.5×]) (Wilcoxon rank-sum test: *P* = .00001) (Figure 2*C* and 2*D*). Variants of interest were mostly detected in A, C1, and C1-M27 subclades (Figure 2*B*) in contrast to the C2 subclade where ESBL gene amplification appeared to be the predominant genomic adaptation to ETP exposure.

### The C2/*H*30Rx Strain MB1860 Initial Response to Ertapenem Exposure Is Amplification of IS*26*-Mediated Pseudocompound Transposons

We passaged MB1860 in the presence of ETP within both standard, serial FTP and MFS platforms as a means to model “fast” versus “slow” adaptation to ETP exposure, respectively [22]. We studied the MB1860 strain since it belonged to the subclade with the largest number of non-CP-CR*Ec* isolates (ie, C2/*H*30Rx) and because it contains a chromosomally located pseudocompound transposon (PCTn) harboring *bla*_CXT-M-15_ and *bla*_OXA-1_ genes, which we formerly described as Tn*MB1860* [4]. All 3 biological replicates from the FTP experiments developed high-level (ie, ETP MICs ≥32 μg/mL) ETP resistance (ETP-R) within 9–12 days (Figure 3*A*). Conversely, it took 53 days to adapt MFS populations to ETP concentrations equal to 0.5 μg/mL (Figure 3*B*), which may reflect the stronger selection and slower adaptation within the MFS environment.

Consistent with the data from the mutation frequency analysis, for both the FTP and MFS platforms, we detected *bla*_OXA-1_ and *bla*_CTX-M-15_ amplifications at ETP concentrations as low as 0.06 and 0.01 μg/mL in the FTP and MFS systems, respectively (Figure 4*A* and 4*B*; detailed genomic context of MB1860 PCTn is presented in Supplementary Figure 2). Genome-wide analysis of daily population isolates exposed to 1× ETP MIC (FTP day 2, MFS day 23) showed that, for both the FTP and MFS platforms, gene amplification was focused at PCTn regions (Supplementary Figure 3*A*), with a high degree of modularity with respect to IS*26* elements being amplified (Supplementary Figure 3*B*). For example, in FTP population 1 (P1) and population 3 (P3), we consistently observed amplification of a nearly 60-Kb region (Supplementary Figure 3*B*) starting with the 5′ insertion of the MB1860 PCTn into the *cirA* gene and downstream of another IS*26* element that truncated a DUF-encoding gene (Figure 4*A*). In contrast, for the MFS populations, gene amplification occurred only in the approximately 13 Kb MB1860 PCTn region for MFS-P1 isolates, the whole 60 Kb region for MFS population 2 (P2) isolates, and initially the 60 Kb until the final MFS-P3 day 52 isolate had PCTn amplification only (Supplementary Figure 3*B* and Figure 4*B*). For all isolates, we observed significant amplification of *bla*_CTX-M-15_, up to 20×, whereas *bla*_OXA-1_ was inconsistently amplified both over time, among isolates, and between platforms (Figure 4). Differential amplification patterns could be observed across each of the experimental platforms as well as each of the independent populations, highlighting the dynamic nature of PCTns mediated by IS*26* elements (Supplementary Figure 4*A–E*). Both *bla*_OXA-1_ and *bla*_CTX-M-15_ amplifications were confirmed with FTP and MFS P1 populations using quantitative polymerase chain reaction (qPCR) where we saw consistent differential and concurrent amplifications of each respective AMR gene in each experimental platform consistent with our WGS data copy number variant analysis (see Supplementary Methods and Supplementary Figure 5).

Independent of experimental evolutionary platform, there were only a small number of genetic variations, the majority of which appeared after prolonged, increasing ETP exposure (Supplementary Figure 4*A–F* and Supplementary Table 6). For the FTP strains, variants of interest included nonsynonymous mutations in *ompC* (outer membrane porin C), *ompF* (outer membrane porin F), and *purM*, which encodes a purine metabolism gene (Supplementary Figure 4*D* and 4*E*). For the MFS platform, we identified no variants of interest in daily populations with the exception of a nonsense mutation in *surA* from P3 isolates (Supplementary Figure 4C). There were nonsynonymous mutations in EPI isolates present in genes encoding proteins involved in outer membrane composition such as the histidine kinase regulator of OmpC/F expression, EnvZ (P1), and the previously observed chaperone protein SurA (P2 and P3). Similar *surA* mutations were identified in a subset of our mutant fluctuation assay results described above during initial ETP adaptation.

In light of the recurrent finding of outer membrane protein variation in all our assays, we tested for the presence of the key outer membrane protein OmpC using Western immunoblot as shown in Figure 4*C* and 4*D*. For the FTP-passaged strains, we only detected OmpC at day 2, whereas no OmpC was detected by day 6 even in the absence of genetic mutations affecting *ompC* detected from this daily population (Figure 4*C*; blue box). No OmpC was identified in any of the FTP EPIs consistent with the *ompC* mutations detected in these strains exposed to high ETP concentrations (Figure 4*C*). For the MFS isolates, we consistently detected OmpC production and no *ompC/ompF* mutations were detected; however, the OmpC production was decreased in the MFS-P3-D52 isolate (Figure 4*D*; red box) corresponding to when a *surA* nonsense mutation was detected, as well as the MFS-P3-D22 isolate prior to any detected genomic changes (Figure 4*D*; blue box). This is consistent with reduced expression of OmpC being an important adaptation in response to higher concentrations of ETP.

### Identification of Consistent Impact of IS*26* on the MB1860 Transcriptome in Response to ETP Exposure

To identify genes with altered transcript levels following ETP exposures in both experimental evolutionary systems, we performed RNA-Seq on cells grown with and without ETP exposure (Figure 5). We performed reverse-transcription qPCR validation on 3 target genes (ie, IS*26* transposase gene, *bla*_CTX-M-15_, and *bla*_OXA-1_) and found similar expression patterns for each respective experimental evolutionary platform (Supplementary Figure 6). A Pearson correlation coefficient heatmap of differential expression data is presented on Supplementary Figure 7. Of the 24 genes with significantly differentially expressed transcript levels following ETP exposure in both platforms (defined as at least absolute value log_2_ fold change >1 and adjusted *P* < .05), 12 were genes that were harbored within the MB1860 PCTn including *bla*_OXA-1_ (Figure 5*A*) and *bla*_CTX-M-15_ (Figure 5*A* and 5*B*) genes (Supplementary Table 7). Indeed, of all the genes with increased transcript levels in both systems, *bla*_CTX-M-15_ had the highest (in the FTP) and second highest (in the MFS) normalized read counts, indicating the marked transcriptional output dedicated to *bla*_CTX-M-15_ following ETP exposure (Supplementary Table 7). When performing gene set enrichment analysis, the one activated gene set activity conserved across both platforms was “transposase activity” (Figure 5*C* and 5*D*). The gene set fraction was <30% for each group and largely confined to ISs (eg, IS*26*) that were bracketed by the MB1860 PCTn (Supplementary Table 7).

## DISCUSSION

There has been recent research characterizing the predominant carbapenem resistance mechanisms by which *E coli* either acquire carbapenemases through horizontal gene transfer or increase production of β-lactamase–encoding genes and concurrently reduce cellular permeability [34]; however, there has remained a gap in knowledge with regard to how high-risk ESBL-*Ec* adapt to initial carbapenem exposure. Our study objective was to comprehensively investigate the genomic and transcriptomic responses to initial carbapenem exposure using ETP selection with the successful extraintestinal pathogenic *E coli* ST131 lineage as a model to determine evolutionary pathways. Using multiple complementary strategies, we found that IS-mediated gene amplification is the predominant ST131 adaptation to ETP exposure, which can potentially progress to non-CP-CR*Ec* development.

A key finding of our study was identification of IS-mediated amplification of ESBL-encoding genes as a primary ESBL ST131 adaptive response, particularly for C2/*H*30Rx strains (Figure 2). Our comparative genomics analysis of mutants recovered from our fluctuation assay indicated that ST131 mutants consistently either initially responded to ETP exposure by increasing ESBL gene copies or disrupting porin production (Figure 2*C* and 2*D*). Multiple studies have shown how *E coli* and other *Enterobacterales* spp modulate their outer membrane permeability to decrease carbapenem influx into the periplasm and concurrently increase expression of ESBL- or pAmpC-encoding genes [6, 35–38]. Nevertheless, both reduced porin-mediated permeability [39] and gene amplification in the absence of selection [40, 41] have been shown to impart significant fitness costs, potentially limiting transmission. A recent case series study of a clinical *E coli* isolate demonstrated that despite high PCTn-mediated chromosomal amplification of *bla*_TEM_, this particular isolate did not incur significant fitness costs [42]. Furthermore, recent studies from different geographic locales [43–45] have found, consistent with our study, a high prevalence of the ST131 C2/*H*30Rx subclade with stable chromosomal PCTn incorporation harboring the *bla*_CTX-M-15_ gene in the absence of IncF type plasmid carriage. These results suggest chromosomal integration may impart a survival benefit to ameliorate gene amplification–incurred fitness costs that arise from plasmid-mediated *bla*_CTX-M-15_ amplifications. However, increased plasmid-mediated amplification of *bla*_CTX-M-27_ with elevated ETP MIC fold changes in C1-M27 mutant progeny (Figure 2*A* and 2*B*) illustrates the potential for multiple, successful evolutionary pathways toward ETP-R development.

That porin mutation imparts a significant fitness cost was reflected in the complex pattern by which the C2/*H*30Rx MB1860 strain altered porin function as detected in our experimental evolution platforms. In both the FTP and MFS platforms, decreased OmpC production at the protein level was not detected until several days after ESBL gene amplification at a time when no genomic mutations were detected (Figure 4). The amount of outer membrane porin protein content in the outer membrane can be affected at the gene (eg, insertions/deletions [5]), transcription (eg, 2-component signal transduction systems, EnvZ/OmpR [6, 46]), posttranscriptional (eg, MicF [47]), and even posttranslational level (eg, chaperone proteins like SurA [32]). We did not identify any significant difference in porin gene transcript levels following ETP exposure, but this analysis was only performed at a single time point and thus cannot entirely exclude a transcriptional-level explanation for the initial OmpC protein changes. For the MFS platform, we did not identify *ompC* or *ompF* mutations, but rather there were mutations in *envZ* and *surA*, which was reflected in the decreased but still detectable amount of OmpC protein. This suggests that the fitness cost imparted by *ompC* or *ompF* mutation was too severe under the MFS conditions to become stable in the population, suggesting that weaker alleles were more successful at low ETP concentration; for example, weaker alleles were sufficient to balance fitness costs against benefits at lower concentrations of ETP. Conversely, in the more nutrient-rich FTP platform, inactivating mutations in *ompC* and *ompF* became detectable once ETP-exposed passaged populations of MB1860 crossed the ETP-R breakpoint, consistent with these 2 necessary mechanisms contributing to non-CP-CR*Ec* development observed in our clinical isolates.

Our study has a few limitations including small sample size and limited characterization of other evolutionary trajectories under alternative carbapenem drug exposures. Nevertheless, our results indicate a robust response to ETP selective pressure across all ESBL-positive ST131 subclades we tested with repeat measures of initial genomic (eg, ESBL amplification or Omp mutation) and transcriptomic (eg, transposase activity) responses, which were consistent with mechanisms we detect in MEM-resistant clinical isolates [5]. Future work will be necessary to determine upstream targets of gene amplification and how initial carbapenem adaptations contribute to noncanonical resistance mechanisms such as heteroresistance in the clinical setting [48–50].

In summary, we have used experimental evolutionary platforms with complementary multiomic approaches to comprehensively assess the initial adaptation and progression of carbapenem resistance within ST131 *E coli* bacteremia isolates. Our findings support unique evolutionary trajectories within ST131 subclades with the predominant ST131 C2/*H*30Rx subclade initially responding to carbapenem selection through IS*26*-mediated amplification of ESBL-encoding genes in the absence of detected genomic changes affecting outer membrane permeability. Given the emerging understanding of the role of gene amplification in resistance development to multiple classes of antibiotics, increased understanding of gene amplification regulation could lead to new preventive or treatment strategies.

## Supplementary Data

Supplementary materials are available at *The Journal of Infectious Diseases* online (http://jid.oxfordjournals.org/). Supplementary materials consist of data provided by the author that are published to benefit the reader. The posted materials are not copyedited. The contents of all supplementary data are the sole responsibility of the authors. Questions or messages regarding errors should be addressed to the author.

## Notes

***Acknowledgments.*** We would like to thank the University of Texas MD Anderson Cancer Center (MDACC) clinical microbiology laboratory staff for their hard work in identifying, handling, and transferring these pathogenic strains of interest to us for our research projects. The authors acknowledge the support of the High-Performance Computing research facility at MDACC for providing computational resources that have contributed to the research results reported in this paper.

***Ethics statement.*** This study has received ethical approval through the MDACC Institutional Review Board (Protocol ID: PA13-0334).

***Financial support*.** W. C. S. was supported through the National Institute of Allergy and Infectious Diseases (NIAID) T32 AI141349 Training Program in Antimicrobial Resistance. S. S. A. was supported by a Dell Family Fund for the School of Health Professions scholarship. The research in the A. Ko. laboratory is supported by the National Institute of General Medical Sciences (grant number R01GM133904) and the Welch Foundation (research grant number AU-1998-20220331). C. A. A. is supported by the National Institutes of Health (NIH)/NIAID (grant numbers K24AI121296, R01AI134637, R01AI148342–01, and P01AI152999). Support for this study was also provided by NIAID (grant numbers R21AI151536 and P01AI152999 to S. A. S.). Core grant CA016672 (Advanced Technology Genomics Core [ATGC]) and NIH 1S10OD024977-01 grant provide funding for the ATGC sequencing facility at MDACC.
