# Supplementary material for: Comprehensive Assessment of Initial Adaptation of Extended-Spectrum β-Lactamase–Positive ST131 Escherichia coli to Carbapenem Exposure
Source: J Infect Dis. 2024 Nov 27;231(4):e685–96. doi: 10.1093/infdis/jiae587 (PMC11998557; doi:10.1093/infdis/jiae587)
Supplement: jiae587_Supplementary_Data [file jiae587_supplementary_data.zip › SUPPLEMENTAL METHODS_revision_clean.docx]

**SUPPLEMENTAL METHODS**

*Modified Luria Delbruck assay*

Briefly, plate swabs (3-5 colonies) were subcultured in Mueller Hinton Broth (MHB), grown to mid-log phase (~0.5 OD_600_), and then diluted down to a 0.5 MacFarland standard. Cells were then inoculated at baseline to ~100 cells/well and grown in MHB for 3 hours at 37°C in 2 96-well plates (192 wells total). After 3 hours elapsed, carbapenem was added at ~0.75× MIC and samples were grown for 20 hours at 37°C. Ten μL was aliquoted from a random well prior to carbapenem exposure and serially diluted out on MHA plates for colony count enumeration. The BioTek Synergy HTX Multimode plate reader was used to measure optical density (600 nm) with Gen5.3.10 software. Wells with OD_600_ >=0.1 measurements after 20 hours were classified as mutants. Ten μL of culture from positive wells are then re-inoculated with ~0.6× carbapenem MIC to confirm mutant growth. Mutation frequency is then calculated as follows: $Mutation freq per {10}^{8}cells =1-\sqrt[c]{\frac{w_{e}}{w_{t}}}\times1e8$

where c = cells/well; w_e_ = empty wells; w_t_=total wells.

The limit of detection (LOD) was calculated as follows: $LOD=\left( 1-\left( \frac{w_{t-1}}{w_{t}} \right)^{\frac{1}{c}} \right)\times{10}^{8}$ with LOD varying as a function of cells/well calculated at administration of antibiotic and total wells (which was 192). Ten μL of culture from positive wells were then re-inoculated with ~0.75× carbapenem MIC to confirm mutant growth.

*Microfluidic system and flask transfer experimental evolutionary platform protocols*

Briefly, samples are grown overnight in LB, and samples are standardized to 2.5e7 CFU/mL prior encapsulation into microdroplets. Additionally, aqueous (30 μL/min) and oil (100 μL/min) phase flowrates were adjusted to achieve a desired lambda = 10 (cells/microdroplet). After incubation overnight at 37^o^C, the cells were decapsulated, quantitated and then re-encapsulated at ~10 cells/microdroplet again to complete one cycle of experimental evolution. ETP concentration was gradually increased to produce a very weak selection gradient from 0 to 0.5 ug/ml ETP over 53 days. For the standard, batch culture, flask transfer protocol (FTP), individual MB1860 colonies that were streaked out on LB agar were grown up in triplicate overnight in LB broth, incubated at 0.5×, 1×, 1.5×, 2× ETP MIC and incubated at 37ºC 250 RPM. Next day isolates with highest observable growth were then passaged at until growth was observed at the end point ETP concentration (32 μg/mL ETP). Each MFS passage/iteration was approximately 48 hours (*e.g.,* Day 22 isolates would be equal to the 11^th^ passage/iteration) whereas each Flask Transfer Protocol (FTP) passage was equal to 24 hours.

*RNA-Seq*

Three mL of each sample was treated with RNAprotect (QIAGEN) following manufacturer’s instructions, and pellets were stored at 80°C until each sample and respective replicate was ready for RNA extraction. RNA was extracted using the QIAGEN RNeasy Mini Kit using manufacturer’s instructions. RNA-seq was performed on an Illumina NovaSeq6000 instrument.

*Comparative Genomics*

The filtered polymorphic sites output from Gubbins was used as input to the hierBAPS (rhierbaps-v1.1.4) function to reveal subclade structure [1] using default parameters and level 2 as subclade assignment. Comprehensive ST131 subclonal genomic characterization was performed using the ST131typer command-line tool [2]. AMRFinderPlus (v3.11.26) was performed to determine both AMR and virulence factors identified in both short- and long-read assemblies using database version 2024-01-31.1. Following variant calling analysis using Snippy-v4.6.0 as described in the main methods section, nonsense, frameshift mutations, and missense mutations were characterized in genes that directly or indirectly affect Omp gene activity. To analyze the impact of detected missense mutations, we used the wild-type FASTA amino acid sequences for the proteins of interest. 3D structural predictions were generated using Phyre2, which models protein structures based on homologous templates [3]. We then applied the Missense3D tool to assess the potential structural impact of each mutation, predicting whether it would result in structural damage [4]. Convict-v1.0 was used to estimate AMR gene copy numbers as previously described [5].

*Differential Expression Analysis*

BBDuk was used to trim Nextera Illumina adapters from RNA paired-end reads using default parameters with modifications [6]. Fast-QC-v0.12.1 [7] was used to perform quality control on trimmed RNA reads. One sample (MFS-P1-E3 = Experimental sample 3) was censored due to low read quality metrics based on Fast-QC output. Trimmed RNA-reads were aligned to MB1860 (RefSeq #: NZ_CP049085.2) using the STAR aligner [8]. Htseq was used for enumerating read counts of uniquely mapped reads [9]. The R package DESeq2 (v1.42.0) was used for differential gene expression analysis [10]. Median of ratios was used to normalize transcripts and log2 fold change were shrunk to stabilize for variance and low read counts. Gene set enrichment analysis was performed on DESeq2 output with the R package ClusterProfiler (v4.10.0) [11].

*DNA (qPCR) and RNA (qRT-PCR) quantification*

To validate our *in silico* copy number variant (CNV) quantification, we chose MB1860 strains from the FTP (FTP-P1-D9) and MFS experimental platform (MFS-P1 Day 6 10 and 52) respectively for qPCR analysis. Each time point was cultured in triplicate on at least two separate days (for a minimum of six biological replicates) to mid-exponential phase (OD600 ~ 0.5) in LB broth (ThermoFisher) at 37°C with shaking at 220 rpm. DNA was isolated using the DNEasy kit (Qiagen), and qPCR was conducted with TaqMan reagents on the StepOne Plus Real-Time PCR platform (Applied Biosystems).

To validate RNA-seq differential expression analysis, we performed RNA transcript level analysis, derived from the FTP and MFS biological replicates described in the above RNA-seq section above. RNA was extracted from the cell pellets using the RNEasy kit (Qiagen) and then converted to cDNA using the High-Capacity cDNA Reverse Transcription Kit (Applied Biosystems). Relative transcript levels of the β-lactamase-encoding genes (*bla*_OXA-1_ and *bla*_CTX-M-15_) and IS*26* transposase gene were measured using TaqMan reagents on the StepOne Plus Real-Time PCR system (Applied Biosystems). Transcript levels of aforementioned target genes were normalized to the endogenous control gene *rpsL* using the ΔCt method. Primers and probes for qPCR and qRT-PCR are listed in Table S8.

**REFERENCES**

1. Tonkin-Hill G, Lees JA, Bentley SD, Frost SDW, Corander J. RhierBAPS: An R implementation of the population clustering algorithm hierBAPS. Wellcome Open Res **2018**; 3:93.

2. Johnston BD, Gordon DM, Burn S, et al. Novel Multiplex PCR Method and Genome Sequence-Based Analog for High-Resolution Subclonal Assignment and Characterization of Escherichia coli Sequence Type 131 Isolates. Microbiology Spectrum **2022**:e01064-22.

3. Kelley LA, Mezulis S, Yates CM, Wass MN, Sternberg MJE. The Phyre2 web portal for protein modeling, prediction and analysis. Nature Protocols **2015**; 10:845-58.

4. Ittisoponpisan S, Islam SA, Khanna T, Alhuzimi E, David A, Sternberg MJE. Can Predicted Protein 3D Structures Provide Reliable Insights into whether Missense Variants Are Disease Associated? J Mol Biol **2019**; 431:2197-212.

5. Shropshire W, Konovalova A, McDaneld P, et al. Systematic Analysis of Mobile Genetic Elements Mediating β-Lactamase Gene Amplification in Noncarbapenemase-Producing Carbapenem-Resistant Enterobacterales Bloodstream Infections. Msystems **2022**; 7:e00476-22.

6. Bushnell B. BBMap: a fast, accurate, splice-aware aligner: Lawrence Berkeley National Lab.(LBNL), Berkeley, CA (United States), **2014**.

7. Andrews S. FastQC: a quality control tool for high throughput sequence data: Cambridge, United Kingdom, **2010**.

8. Dobin A, Davis CA, Schlesinger F, et al. STAR: ultrafast universal RNA-seq aligner. Bioinformatics **2013**; 29:15-21.

9. Anders S, Pyl PT, Huber W. HTSeq--a Python framework to work with high-throughput sequencing data. Bioinformatics **2015**; 31:166-9.

10. Love MI, Huber W, Anders S. Moderated estimation of fold change and dispersion for RNA-seq data with DESeq2. Genome Biology **2014**; 15:1-21.

11. Wu T, Hu E, Xu S, et al. clusterProfiler 4.0: A universal enrichment tool for interpreting omics data. Innovation (Camb) **2021**; 2:100141.
