# Supplementary material for: Comprehensive Assessment of Initial Adaptation of Extended-Spectrum β-Lactamase–Positive ST131 Escherichia coli to Carbapenem Exposure
Source: J Infect Dis. 2024 Nov 27;231(4):e685–96. doi: 10.1093/infdis/jiae587 (PMC11998557; doi:10.1093/infdis/jiae587)
Supplement: jiae587_Supplementary_Data [file jiae587_supplementary_data.zip › Supplemental_figures_revision_clean.docx]

**SUPPLEMENTAL FIGURES**


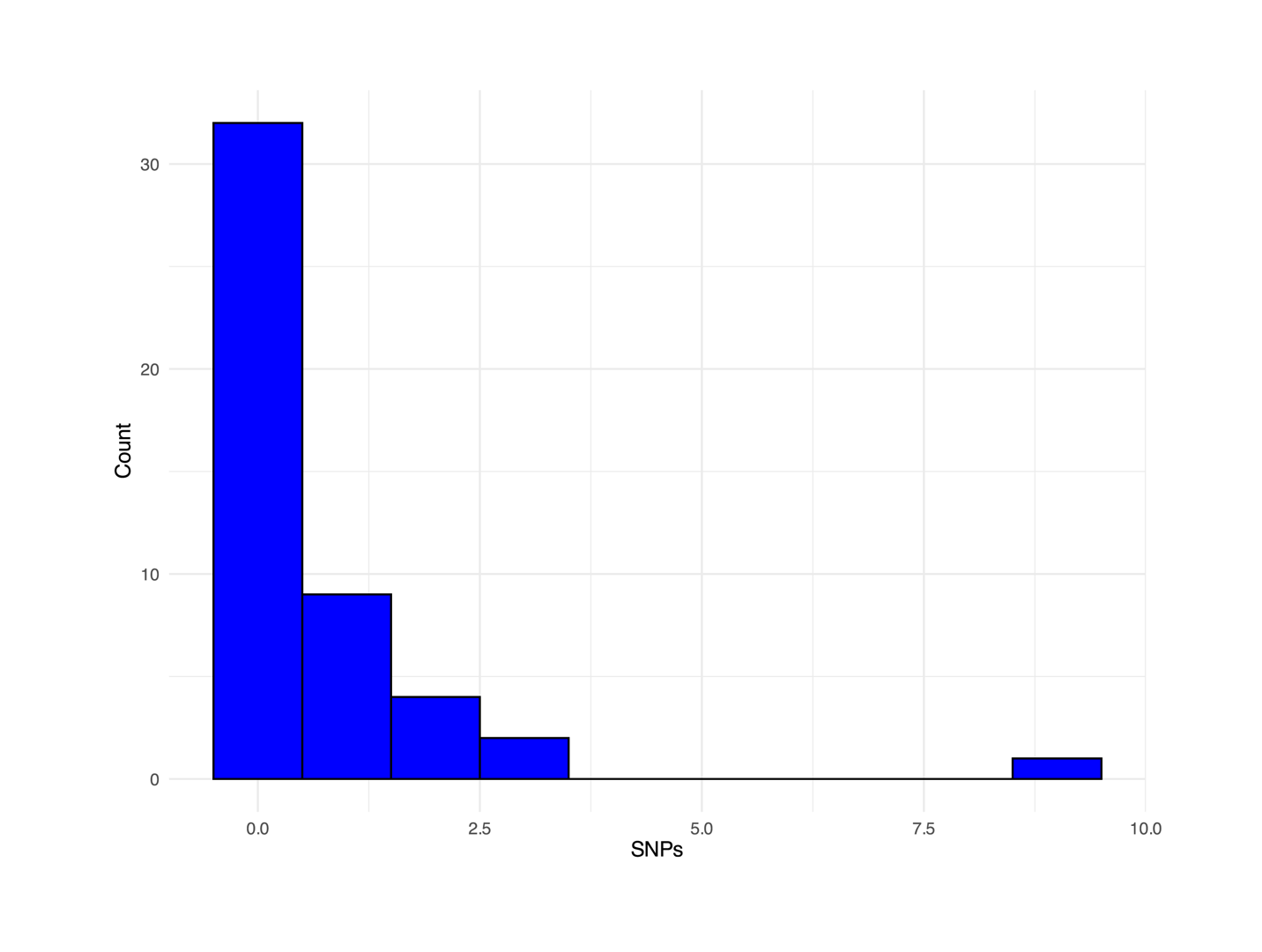


**Fig. S1. Histogram of SNPs detected across 48 fluctuation assay mutants.**

**
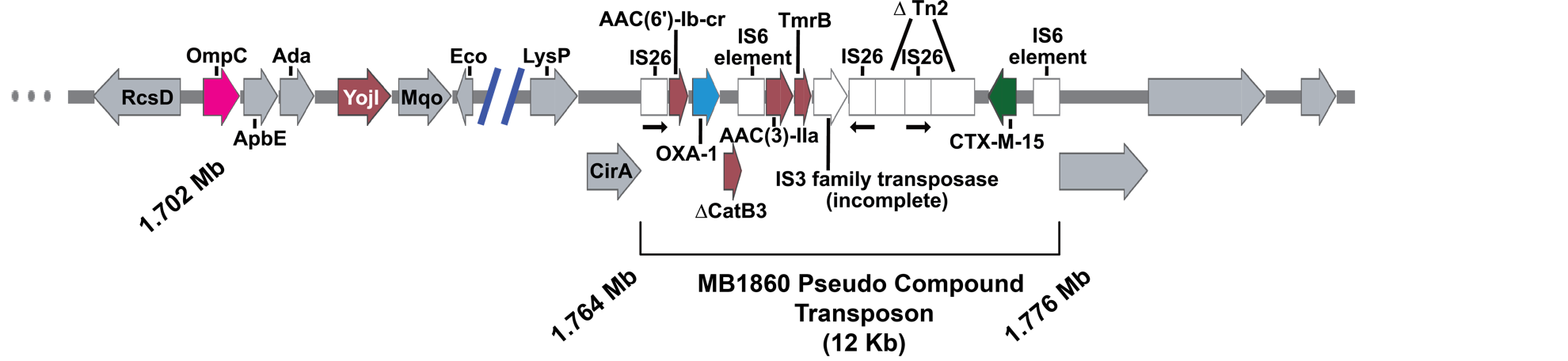
**

**Fig. S2. MB1860 Pseudocompound Transposon.** Presented is the 12 Kb PCTn bracketed by IS*26* elements (white) with *bla*_OXA-1_ (blue) and *bla*_CTX-M-15_ (green) presented. This is an enlarged depiction of MB1860 PCTn as presented in Fig 4AB. The *ompC* gene (pink) is presented here shown ~ 60 Kb downstream of the MB1860 PCTn.

**
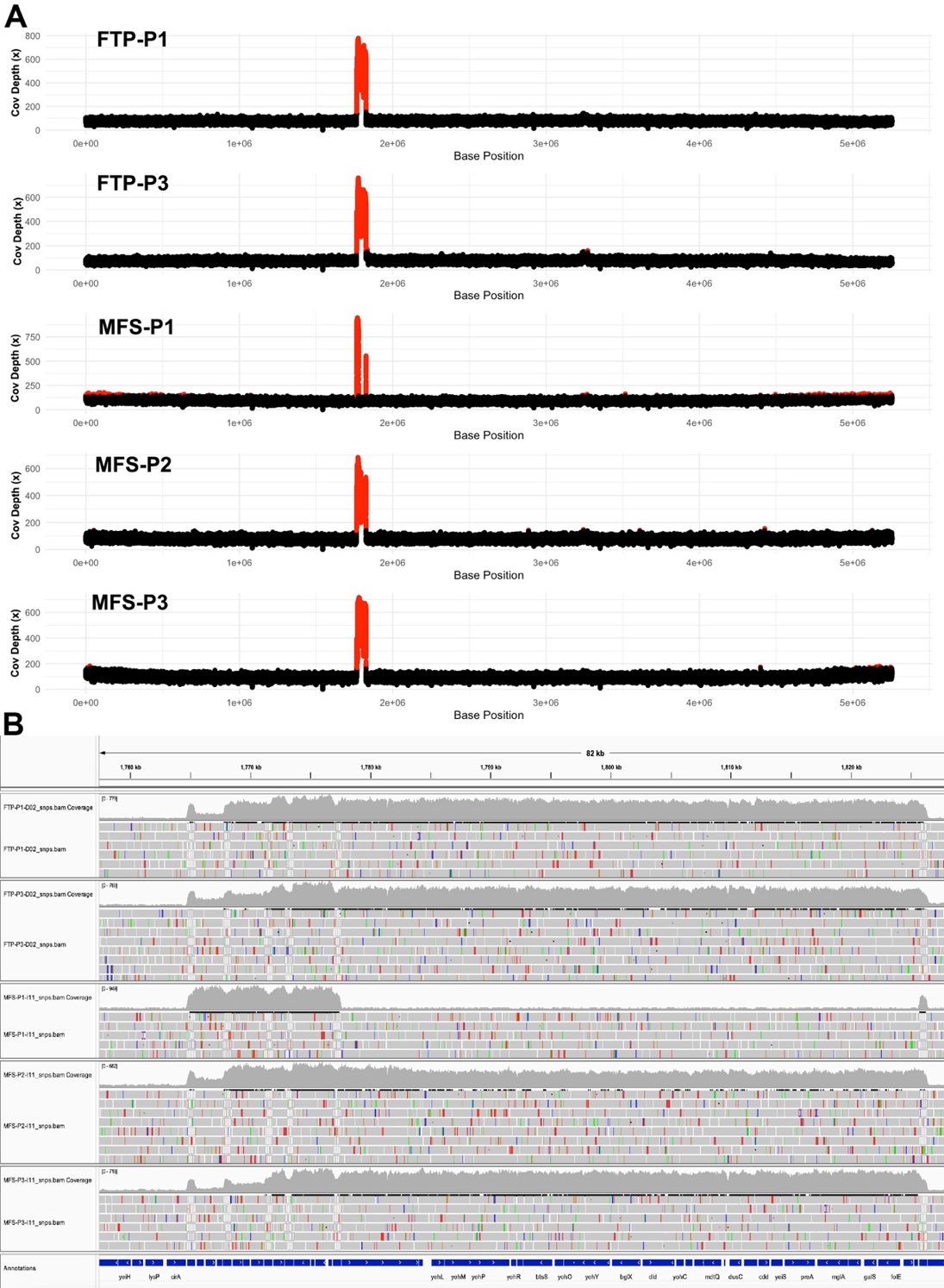
**

**Fig. S3. Chromosome-wide coverage depth for FTP and MFS daily populations. (A)** Genome wide coverage of MB1860 (5 Mb) of FTP and MFS daily populations collected at 1x ETP MIC exposure (Day 2 and Day 22 respectively). Red regions indicate where coverage depth exceeded mean coverage + 1.5 standard deviations. Large red peak for each population corresponds to MB1860 PCTn **(B)** Coverage focused on region corresponding to MB1860 PCTn that is notably increased in **(A)**.

**
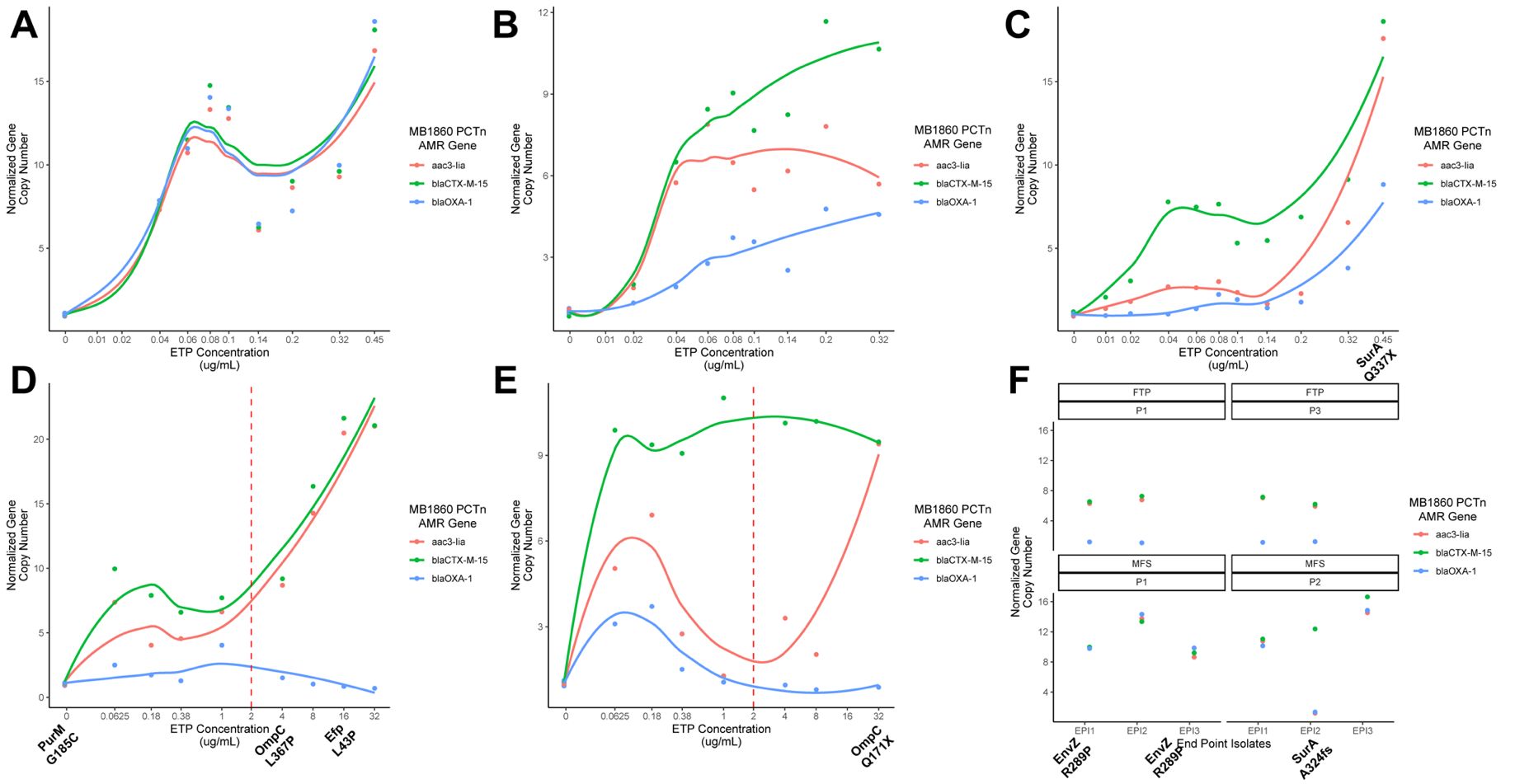
**

**Fig. S4. Copy number variation of AMR genes located within MB1860 pseudocompound transposon from daily population and end-point isolates. (A-C)** MFS populations (P1-3 respectively) and **(D-E)** FTP populations (P1 and P3 respectively) with variants of interest indicated at ETP exposure they were first detected. Loess curves are included to indicate trend of AMR encoding gene copy number trends. Vertical dotted red line in **(D)** and **(F)** indicate CLSI breakpoint for ETP-R. **(F)** Copy number variation of end point isolates (EPIs) selected from the daily population with last ETP exposure. Note that FTP P3 daily population and MFS P3 EPIs were not available for sequencing respectively.


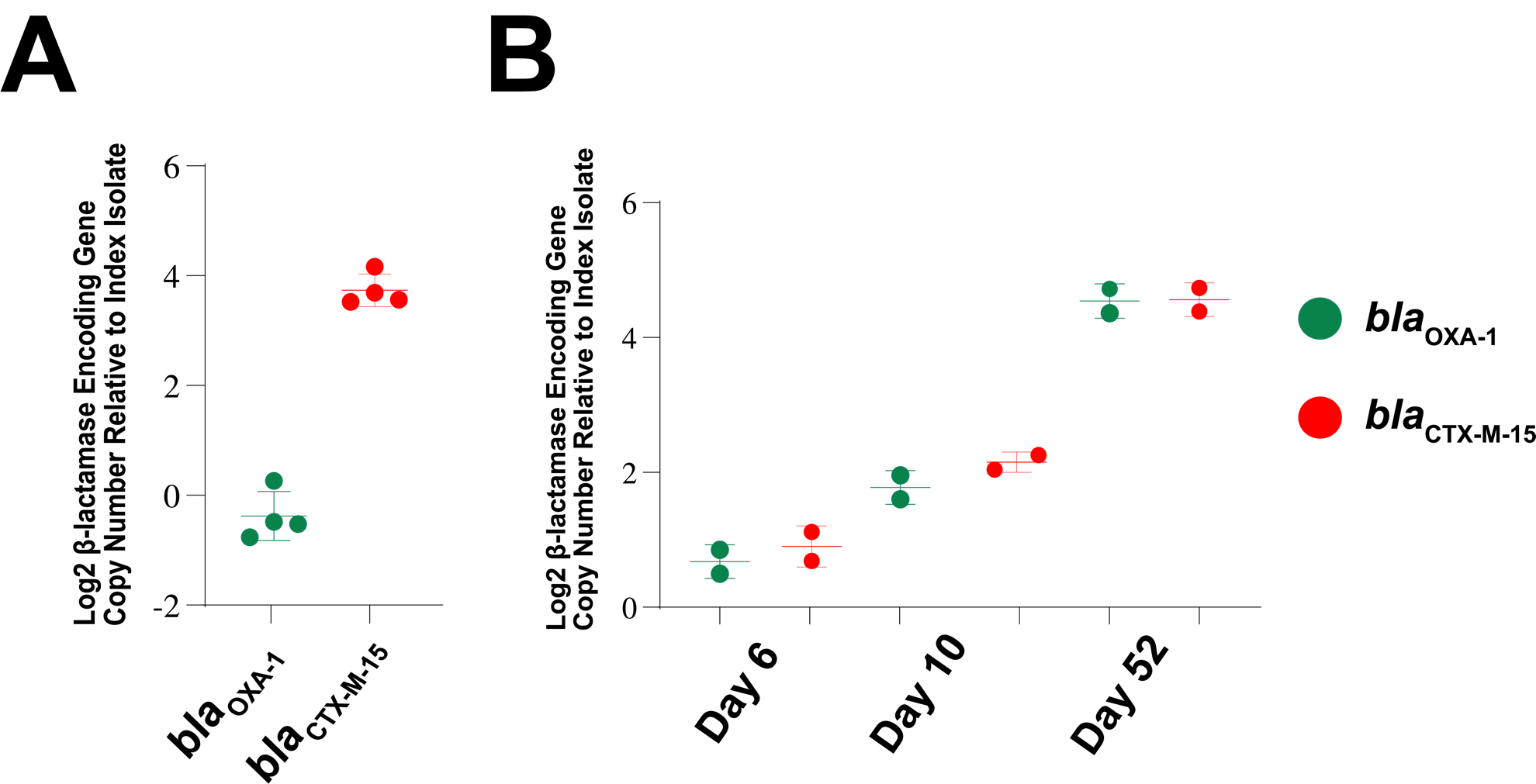
 **Fig. S5. qPCR Quantification of *bla*_OXA-1_ and *bla*_CTX-M-15_ gene copy numbers. (A)** Single time point for flask transfer protocol (FTP) Population 1, Day 12 (*i.e.*, ETP exposure 32x MIC) for *bla*_OXA-1_ (green) and *bla*_CTX-M-15_ (red) respectively. **(B)** qPCR analysis for day 6, 10, and 52 (ETP exposure 0.2X, 0.5X, and 7.5X MIC) for *bla*_OXA-1_ (green) and *bla*_CTX-M-15_ (red) respectively.


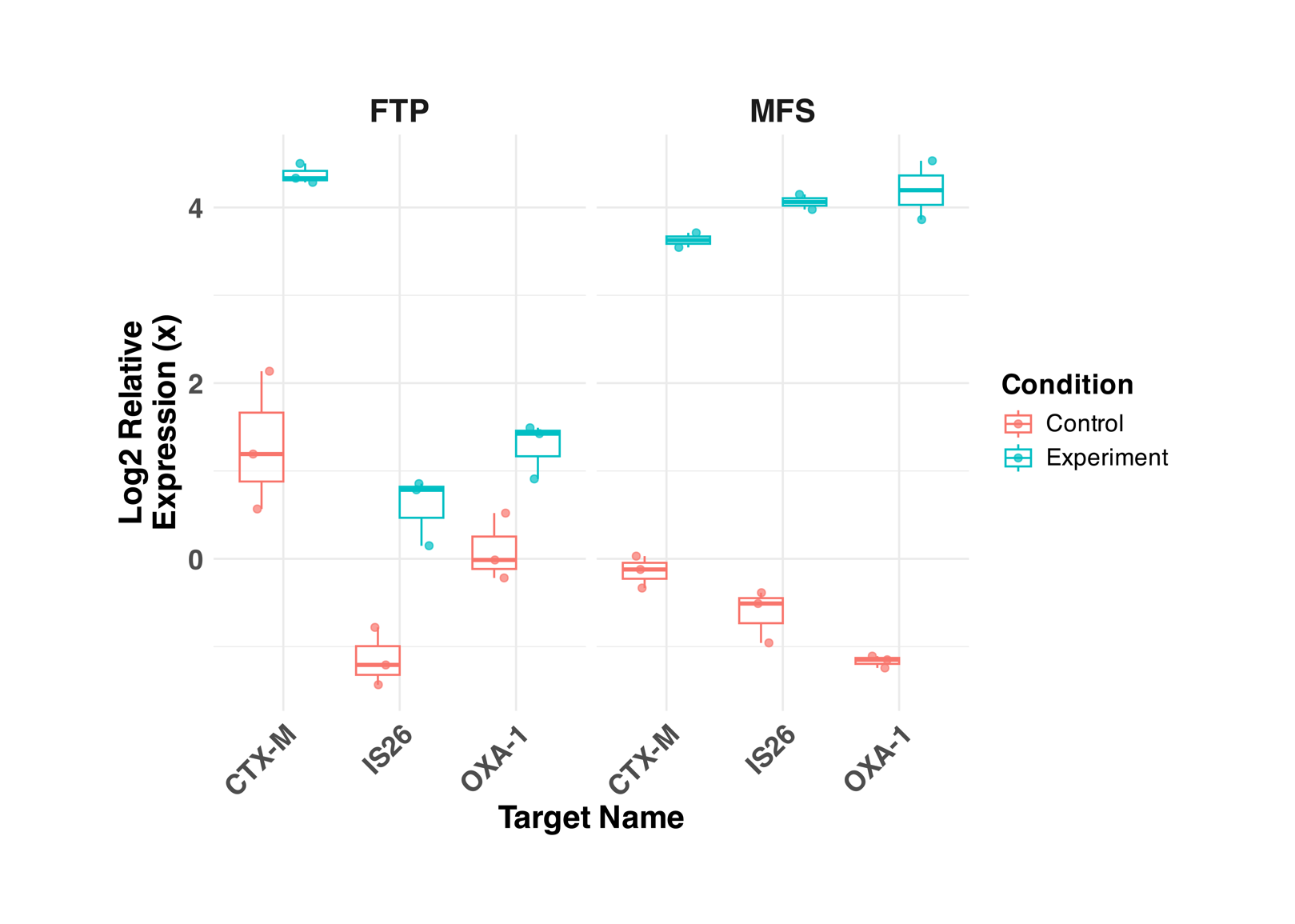


**Fig. S6. RNA-seq Validation using qRT-PCR.** Scatterplot of FTP (left) and MFS (right) gene expression (Log 2 relative expression) of three target genes using *rpsL* as endogenous control gene. Condition (Control = Passage control; Experiment = 1X ETP exposed isolate) labelled in legend.

**
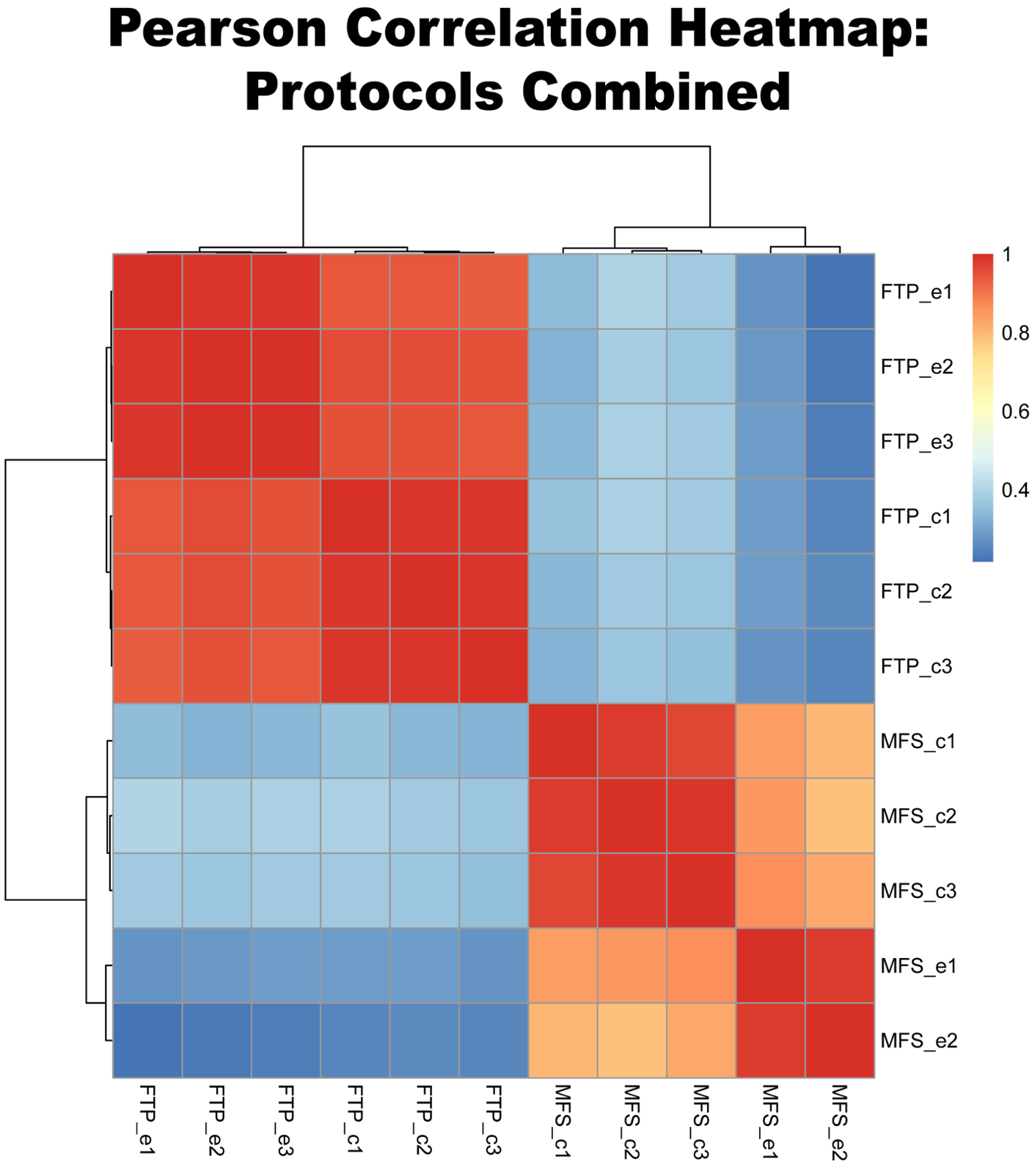
**

**Fig. S7. Correlation heatmap of differential expression observed in FTP and MFS platforms.** Suffix following FTP or MFS with ‘e’ or ‘c’ indicates ‘experiment’ or ‘control’ respectively with replicate number.
